# Editorial: New hypervalent iodine reagents for oxidative coupling—volume II

**DOI:** 10.3389/fchem.2022.995702

**Published:** 2022-09-05

**Authors:** Toshifumi Dohi, Jian-Wei Han, Ravi Kumar

**Affiliations:** ^1^ College of Pharmaceutical Sciences, Ritsumeikan University, Shiga, Japan; ^2^ School of Chemistry and Molecular Engineering, East China University of Science and Technology, Shanghai, China; ^3^ Department of Chemistry, J. C. Bose University of Science and Technology, YMCA, Faridabad, India

**Keywords:** hypervalent compounds, iodine, reagent, oxidative coupling, synthetic application

Hypervalent iodine compounds (HICs), of which iodine is in high oxidation states (+3 or +5), have aroused widespread interest in the fields of synthetic chemistry, and they are valuable as versatile reagents in organic chemistry. Specifically, efficient syntheses make the hypervalent iodine reagents (HIRs) all more practical and environmentally benign.

Historically, the early HICs date back to the 1880–1890s, when Willgerodt prepared (dichloroiodo)benzene as the first HIC, followed by the discovery of (diacetoxyiodo)benzene, iodosylbenzene, 2-iodoxybenzoic acid (IBX) and diaryliodonium salts successively. These early HICs are very useful reagents that we still frequently use in organic synthesis nowadays. With these structurally diversified HICs in hand, the synthetic utilization of them opened up new synthetic pathways, and then have achieved considerable attention. During the long history of research, exploration of diversification of structures and reactivity with hypervalent iodine chemistry has already led to many useful methodologies for modern organic synthesis. Particularly, the unique reactivity pattern of HIRs is similar to that of the transition-metal species, and the reactions of HIRs are commonly discussed in terms of oxidative addition, ligand exchange, reductive elimination, ligand coupling and even the radical process, which represents the most important hotspot in current organic chemistry. Therefore, renewed interest in recent years on the hypervalent reagents is leading to a boom in new synthetic methodologies, mechanistic understandings, potential applications in bioactive molecules.

Last year in 2021, Toshifumi Dohi (Ritsumeikan University, Japan), Jian-Wei Han (East China University of Science and Technology, China) and Ravi Kumar (J.C. Bose University of Science and Technology, India) have organized a Topic issue of Frontiers in Chemistry that includes 10 articles covering key topics of hypervalent iodine chemistry. The issue was successful to reach 37,044 views so far. Considering the explosive accomplishment in organoiodine chemistry, the managing editor requested to organize the second issue on this topic. We therefore embarked on this Research Topic collection titled “New Hypervalent Iodine Reagents for Oxidative Coupling -Volume II”. It is hoped that the collective efforts of the authors of these articles will serve to broaden the foundation that supports future growth in hypervalent iodine chemistry and the influx of new ideas that growth brings. The articles that make up this topic are contributed by authors from the international community, showcasing the widespread adoption of HICs and their continuous development in synthetic organic chemistry. We introduce the key articles below.

Utilization of HICs and iodine as a versatile combination to produce *O*-radicals from different substrates, such as, alcohols, acetals, and acids is explored in literature. These initially generated *O*-radicals are reported to undergo *β*-scission giving rise to *C*-radicals that readily gets oxidized to cationic intermediates**.** Cationic intermediates, thus formed, can be easily attacked by diverse nucleophiles that give an excellent metal-free synthetic protocol to access various bioactive or potential bioactive targets. Boto et al. presented a selection of one-pot scission-oxidation-addition of *C*, *N*, and *P* nucleophiles involving scission-alkylation, scission-arylation, fragmentation-Diels Alder and other inter- and intramolecular cyclization processes, scission-Ritter, fragmentation-addition of nucleobases, and scission-phosphorylation. The majority of the substrates discussed by the group are amino acids, β-hydroxy amines or carbohydrates.

HICs have emerged as resourceful environmentally benign alternatives to heavy-metal oxidants. However, the common preparation methods of HIRs often require hazardous or expensive oxidants. Hence, direct and sustainable methods for their preparation are always in demand. Recently, many researchers have started exploiting the use of electrochemical oxidation for the preparation of HIRs via the anodic oxidation of iodine(I) precursors. Several oxidative transformations have been successfully achieved by using *in situ* electrogenerated HIRs as reviewed recently by Francke and Wirth research groups (Francke et al., 2018; lsherbini and Wirth, 2018; Francke, 2019; Francke, 2021). Chen and Yang presented an overview of the recent advances in this area during the past 3 years. This minireview encompasses the recent developments of electrochemical synthesis of HIRs and, applications of *in-situ* electrochemically generated HIRs as redox mediators in organic electrosynthesis.

Direct or late-stage incorporation of thio/selenocyanato functional groups is of utmost importance as evident from huge biological applications bearing these moieties. In this regard, Du et al. reported an excellent example of a metal-free hypervalent iodine-mediated electrophilic thio/selenocyanation approach for the construction of functionalized isocoumarin frameworks. The group successfully achieved the synthesis of *C*4-thio/selenocyanated isocoumarins *via* thio/selenocyanation of alkynes using PhICl_2_ and NH_4_SCN/KSeCN reagents. The developed protocol involves the thio/selenocyanation, enabled by thio/selenocyanogen chloride generated *in-situ*, followed by an intramolecular lactonization. A Gram scale synthesis of xyridin A and antitumor activity of the synthesized compounds are also presented.

Another example of hypervalent iodine-mediated alkyne functionalization was presented by Liu et al., in which the group reported chemoselective halogenation of terminal alkynes. The terminal alkynes have been reported to give mono-bromination (1-bromoalkynes), di-bromination (1,2-dibromoalkenes) and, tetra-bromination (1,1,2,2-tetrabromoalkanes) of terminal alkynes using TBAB/PIDA, NaBr/PIDA and NaBr/PIDA (in excess), respectively. A certain amount of water with NaBr/PIDA system changes the dibromination product formation to α,α-dibromoketones.

Liu et al. reported mechanism-dependent selectivity in fluorocyclization of unsaturated carboxylic acids or alcohols by hypervalent iodine. Density functional theory (DFT) studies were performed to understand the unprecedented difference between 6-*endo* and 5-*exo* selectivity in hypervalent iodine(III)-promoted fluorocyclization of unsaturated carboxylic acids or alcohols by difluoroiodotoluene. The research provids insights into the mechanism-dependent selectivity should help advance the development of fluorocyclization reactions with HIRs.

HICs have evolved as mainstream reagents particularly due to their excellent oxidizing ability, high electrophilicity and versatile reactivity. Singh et al. summarized the recent advances in non-palladium catalyzed oxidative coupling reactions using HIRs. This review article critically summarizes the recent developments in non-palladium-catalyzed oxidative coupling reactions mediated by HIRs with great emphasis on understanding the mechanistic aspects in detail.

Goreshnik et al. described oxidation of iodine to dihaloiodate(I) salts of amines with hydrogen peroxides and their crystal structures. In this research article, the authors report a general preparation of dihaloiodate salts of heterocyclic amines (tertiary and quaternary) and their structure was determined by NMR and Raman spectroscopy and single crystal X-ray diffraction.

This collection of articles showcases different aspects of hypervalent iodine compounds towards achieving various valuable organic transformations. Irrespective of the fact that there are continuous expansions in this field, there are still many areas yet to be explored. By taking into account the advantages of hypervalent iodine-mediated oxidative coupling reactions, it seems certain that these reactions will be further utilized in the development of sustainable synthetic methods.
